# Helping mentors address scientific communication in STEM research training helps their mentees stay the course

**DOI:** 10.1186/s40594-024-00497-0

**Published:** 2024-08-28

**Authors:** C. Cameron, H. Y. Lee, C. B. Anderson, E. K. Dahlstrom, S. Chang

**Affiliations:** 1 Department of Behavioral Science, The University of Texas MD Anderson Cancer Center, 1515 Holcombe Blvd, Houston, TX 77030, USA.; 2 Cancer Prevention Research Training Program, Division of Cancer Prevention and Population Sciences, The University of Texas MD Anderson Cancer Center, Houston, TX, USA.; 3 Department of Epidemiology, The University of Texas MD Anderson Cancer Center, Houston, TX, USA.

**Keywords:** Scientific communication, Science identity, STEM, Research career, Language variety, Mentoring, Mentor training, Communication skills, SciComm

## Abstract

**Background:**

Scientific communication (SC) has important social-cognitive, behavioral, and career-related benefits for emerging researchers, but both mentors and mentees find development of SC skills challenging. Whether training mentors to effectively mentor development of SC skills could have a meaningful impact on mentees was not clear. The Scientific Communication Advances Research Excellence (SCOARE) project has conducted faculty training workshops in techniques for mentoring SC skills since 2018. To study indirect workshop effects of mentors’ attendance at the SCOARE workshop on their matched PhD and postdoctoral mentees (*N* = 477), we surveyed mentees before and 6 months after their mentors attended and measured their social-psychological and behavioral outcomes. To examine the effectiveness of the workshop and to explore whether workshop effects vary based on mentee demographic characteristics, including home language variety (speaker of standardized English [STE], non-standardized English [NSTE], or another language [L2]), we conducted multilevel models.

**Results:**

After adjusting baseline scores, mentees of mentors who attended SCOARE workshops (W +) were more engaged in speaking activities (*β* = 0 .30, *p* = 0.016), had higher science identity (*β* = 0.20, *p* = 0.048), and were less likely to reconsider their career due to SC skills (β = − 0.39, *p* = 0.004) than mentees in the W− group. Across demographic groups, mentees of mentors who attended SCOARE workshops showed similar improvements in SC outcomes. Post-doctoral mentees, compared to doctoral mentees, had higher science identity and lower intention to pursue a non-research-intensive career. Comparing mentees of the 3 categories of home language variety, both the NSTE and L2 groups, compared to the STE group, were more likely to reconsider their careers due to SC skills and had a higher intention to pursue non-research-intensive careers both at baseline and post-workshop, suggesting the possibility of language background as a barrier to mentee career progression.

**Conclusion:**

Mentor training for SC skill development can improve social-psychological and behavioral outcomes for mentees, including science identity, frequency of speaking, and reconsideration of research careers due to concerns about SC.

## Introduction

The role of mentoring in improving the science, technology, engineering, and mathematics (STEM) trainee experience and in addressing trainee retention in academic research has seen a surge of interest in recent years, reflecting growing recognition that evidence-based, inclusive, and intentional mentoring practice benefits mentees (Atkins et al., 2020; Diggs-Andrews et al., 2021; Fuhrmann, Halme, O’Sullivan, & Lindstaedt, 2011; Griffin, Gibbs, Bennett, Staples, & Robinson, 2015; Hund et al., 2018; Pfund et al., 2016; Trujillo et al., 2015). Moreover, formal mentor-training programs have been shown to result in desired outcomes for mentees and mentors alike (Johnson, 2015; Sorkness et al., 2017; Stelter et al., 2021; Trejo et al., 2022; Weber-Main et al., 2019).

Among the various competencies that mentors of doctoral and postdoctoral mentees strive to foster, scholarly speaking and writing skills, or scientific communication (SC), is widely recognized as essential for career progression. Many graduate STEM programs now require publication of peer-reviewed research articles as a condition of receiving a PhD, and the number of first-authored peer-reviewed publications remains a widely used criterion for hiring and promotion in many research-related positions. Beyond academic research, technology employers place a premium on communication skills (National Association of Colleges and Employers, 2023; Strauss, 2017). A report of employment patterns among recent graduates in life sciences research in the Netherlands identified language and communication skills as the skills most desired by employers for all life sciences research career paths (van Ravenswaaij et al., 2023). Additional studies have emphasized the barriers posed by a lack among STEM graduates of generic or transferable skills, such as communication and teamwork, as opposed to domain-specific skills, such as experimental techniques (Chamorro-Premuzic, Arteche, Bremner, Greven, & Furnham, 2010; Thompson et al., 2018).

Although indispensable, SC skills can be challenging both to acquire and to mentor (Aitchison & Lee, 2006; Kranov, 2009). Despite an abundance of books, applications, and courses for learning generic SC (Day & Gastel, 2006; Sainani, 2012; Zeiger, 2009), the means for developing SC proficiency on a professional and appropriately specialized level are scant. At advanced stages of training, the mentee must master not only the general norms of SC but also the specific usage, vocabulary, and nuances of the discipline they are training in; this process occurs largely within the mentor–mentee relationship (Aitchison & Lee, 2006; Kranov, 2009) rather than through formalized instruction. Few resources are available to support mentors in helping their mentees to develop the more abstract, discipline-specific research products needed to launch a scholarly career, and STEM mentors often struggle in this area, becoming fatigued and frustrated (Aitchison, 2015). Many assume the additional role of grammar and composition tutor or sometimes simply rewrite significant portions of mentee drafts, solving an immediate problem but ultimately impeding the mentee’s acquisition of SC skills.

### Significance of language use and its relationship to identity

SC skill development is widely perceived to be a straight-forward cognitive task, a matter of learning generic rules and conventions from reference books or workshops. On a deeper level, however, personal and professional language use is a fundamental part of social identity construction and frequently although not completely overlaps with ethnicity, socioeconomic status, or geographic origin, or various combinations of them (Bourdieu, 1991; Bucholtz & Hall, 2005; Emerson & Holquist, 1986; Flores et al., 2014; Halliday & Martin, 1993; Lippi-Green, 1997; Phinney et al., 2001). It is the primary mode of signaling to others who we are as well as how we would like to be perceived in any given situation—as bona fide research scholars, for example. We unconsciously or consciously make attributions and judgments about others and who they are as soon as we hear or read their words. Not having mastered the “right” or accepted type of language for a social situation reveals our relative lack of familiarity or experience with it, and hence the legitimacy of our belonging in that situation.

Home language variety (the type of language we were brought up speaking) may thus contribute to impostor feelings in the speaker as well as trigger unconscious or conscious bias among listeners in educational and work settings, due to misplaced associations of speaking style with intelligence, trustworthiness, and social prestige (Adger, Wolfram, & Christian, 2014; Baugh, 2000; Blake & Van Sickle, 2001; Hudley, Mallinson, Berry-McCrea, & Muwwakkil, 2020; Purnell et al., 1999; Rickford & King, 2016). In the STEM research context, having been raised speaking a non-standardized variety of English or speaking a language other than English has been found to be associated with mentee feelings of discomfort in the research environment about their speech variety, even if the mentee is using standardized English (Cameron et al., 2023). These dynamics of language use are little recognized or appreciated by either the public or academicians, including mentors, curriculum developers, editors, and even some language instructors, but mentors and mentees devote so much energy to honing SC skills because they sense the value of talking and writing like a scientist. Using the “right” language connotes authority, knowledge, and power that is accepted intuitively by listeners and allows early-career researchers to be recognized and respected as professionals (Carlone & Johnson, 2007; Griffin et al., 2015; Hudson et al., 2018). Sounding ‘like a scientist’ is important, and mastery of SC skills is central to achieving belonging and credibility in STEM research.

### Mentoring and the development of SC skills

A series of SC studies grounded in a modified Social-Cognitive Career Theoretical model [SCCT (Lent et al., 1994)] found that social-cognitive factors associated with development and mentoring of SC skills influence doctoral and postdoctoral STEM researchers’ intention to remain in a research career. SCCT models use constructs such as self-efficacy (“can I do this?”), outcome expectations (“what will be the result of doing this?”), interest (“am I interested in this?”), and contextual barriers and supports to investigate how career intentions are formed and maintained. Mentees’ intention to remain in a research career was found to be predicted directly and indirectly by self-efficacy for SC, frequency of SC speaking and writing of any kind (refereed or not), SC outcome expectations, and, in a follow-up study, science identity and mentee reports of mentoring practices received for SC (Anderson et al., 2015; Cameron et al., 2015, 2020). Science identity, or the feeling that one belongs in science or is a “science person” (Chemers et al., 2011), is considered to be an important influence on the career intentions and subjective experience of STEM undergraduate (Aikens et al., 2016; Atkins et al., 2020; Estrada et al., 2018; Findley-Van Nostrand & Pollenz, 2017; Hernandez et al., 2017), postbaccalaureate students (Remich et al., 2016; Gazley et al., 2014), doctoral (Carlone & Johnson, 2007; Griffin et al., 2015), and postdoctoral researchers (Griffin et al., 2015; Hudson et al., 2018).

### Development of the SCOARE workshop intervention

Although faculty mentoring practices for SC were found to influence mentee outcomes, the question of whether mentors could be successfully trained in new practices that would foster inclusive SC development remained unanswered. To investigate this possibility, we designed a mentor-training workshop, Scientific Communication Advances Research Excellence (SCOARE, R25 GM125640), which derived its learning objectives from the social-cognitive model’s constructs. The goal of the workshop was to teach participants to mentor SC skills (rather than directly teach or edit SC), with emphasis on addressing social-cognitive and behavioral elements, as part of an engaged mentoring style. The workshop was designed so that participants needed no specific pedagogical, linguistic, or theoretical background knowledge and to minimize burdensome time commitments either for attending the intervention or for applying the skills in practice. SCOARE has been delivered to over 500 mentors in over 50 workshops since 2018, with robust satisfaction, learning, and perceived usability rates (Dahlstrom et al., 2022).

The workshop is delivered in either one 6-h session or two 3-h sessions and is limited to 20 participants to encourage active participation and engagement. Learning objectives include: (1) to understand one’s role and perspective as a mentor versus that of a trainee in SC development; (2) to learn how to set expectations, create structure, and apply strategies to increase trainee engagement in SC development; (3) to learn to deliver useful and appropriate feedback; and (4) to create, adapt, and personalize mentoring strategies to use with mentees.

The curriculum comprises 5 sections. The first addresses the role of language in not just scholarly writing and speaking, but also identity and social setting, contextualizing the role of non-standardized varieties of English as valid and systematic but not the variety typically encountered in academic settings. Next, the research background that grounds the content of the workshop is reviewed and participants complete a case study that raises issues of mentee linguistic self-doubt, academician discrimination, and possibilities for mentors to interpret and engage with such situations. Section 3 delves into language use and how its three modes of writing, formal presenting, and informal speaking, come into play, with informal speaking in the research environment (“speaking up”), playing a much more important role than most mentors are aware of, and representing an opportunity for developing mentee skills and confidence. Section 4 takes a deep dive into SC mentoring strategies that address learning objectives 2 and 3 (creating structure for communication activities, setting expectations, and providing useful feedback). Finally, the workshop concludes with participants building their own mentoring plan for moving forward, based on their insights from the workshop, including identifying strategies they can put into practice with their mentees immediately.

As a theoretical framework, we were guided by Baumrind’s theory of parenting styles (authoritarian, authoritative, permissive, and neglectful; 1978) and Maccoby and Martin’s concepts of demandingness and responsiveness (1983), aiming for an authoritative style balancing responsiveness with demandingness. We added content that would enable mentors to be more intentionally supportive, such as developing an appreciation of the effects of linguistic acceptance (vs. discrimination) and positioning language varieties as valid alternatives that could co-exist rather than as either ‘correct’ or ‘incorrect’. Activities for this included discussion of critical case studies involving mentee language varieties and how to think and talk about them as well as instruction on providing feedback that is specific, positive, and actionable rather than threatening, demoralizing, or vague. (Vagueness has been identified as a significant concern for writing feedback (Bean, 2001)). Such supportiveness could be manifested through having discussions about language variety with the group, providing feedback that would signal to mentees opportunities to expand skills rather than criticism, scaffolding writing tasks, finding ways to provide ‘small victories’ on the path to mastery, and working with mentees to develop critical thinking skills. Besides responsiveness, demandingness is also a hallmark of authoritative style, and content was added to help mentors identify opportunities to provide structure and high expectations, such as techniques for encouraging mentees to speak up more often, showing them how to use resources that guide mentees to find alternative sources of editing before approaching the mentor for feedback, or establishing and keeping deadlines. Because many mentors express uncertainty about giving feedback and making requests assertively, participants are guided through developing their own action plans about whether, when, and how to do so appropriately.

### Research on SCOARE mentee outcomes

**Fig. 1:**
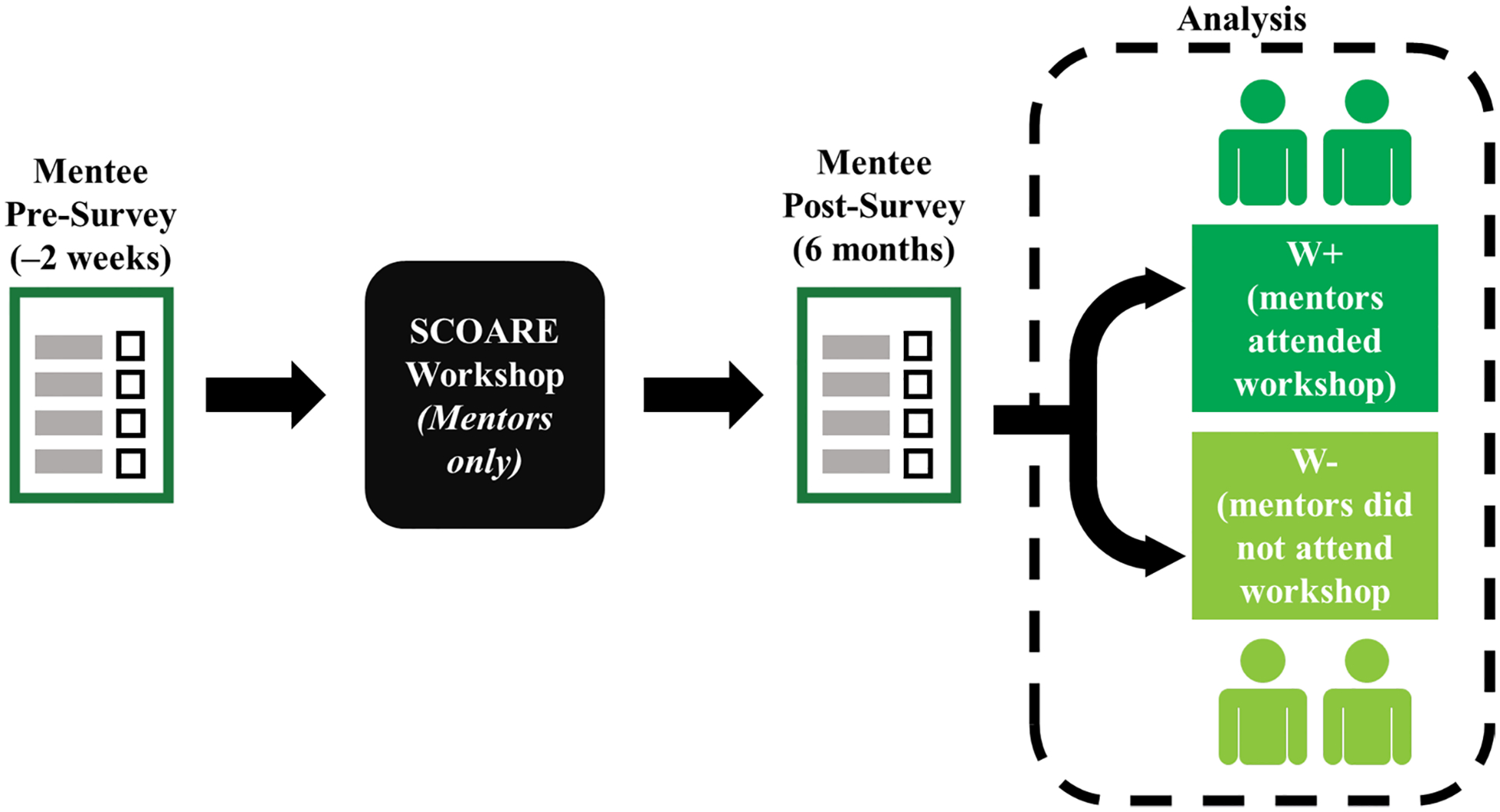
SCOARE study design

To test workshop effects on mentee behavioral and social-cognitive variables (frequency of engaging in SC, science identity, SC self-efficacy, SC outcome expectations, reconsideration of a research career due to SC, and research career intentions) after their mentors participated in the workshop, we conducted a national survey of one group of mentees whose mentors had attended a SCOARE workshop and one group whose mentors had registered but did not attend the workshop. We hypothesized that mentees whose mentors had attended would experience beneficial changes in the variables of interest, compared with mentees whose mentors did not attend. Additionally, we explored whether mentee demographic characteristics were associated with these variables. Figure 1 diagrams the SCOARE study design.

## Methods

### Participants and procedure

Participants were doctoral and postdoctoral mentees of faculty mentors who were recruited to participate in the SCOARE mentor-training study. To recruit the mentors to the workshop and study, we partnered with 4 sites: the University of Colorado, Boulder; Georgia State University; University of Chicago/Big Ten; and the Gulf Coast Consortia in Houston, Texas, comprising 7 biomedical institutions. When the COVID-19 pandemic precluded in-person workshops, SCOARE was converted to a virtual format, which enabled us to offer it to interested research faculty at institutions around the country. STEM faculty mentors at these sites received emails from on-site research administrators inviting them to participate in a 6-h SCOARE SC mentoring skills workshop; mentors from other disciplines expressed interest in participating as well. During registration, mentors were asked to nominate at least one doctoral or postdoctoral research mentee to also participate in the research study but could nominate multiple mentees if they wished. Of the 257 faculty mentors who registered for the research study themselves, and whose nominated mentees also agreed to participate in the study, 220 mentors attended the workshop and 37 did not attend. Non-attendance was typically due to illness, childcare responsibilities, or other last-minute deadlines. There were no differences in mentors’ rank, gender, or race between mentors who attended the workshop and those who didn’t.

Six-hundred twenty-seven nominated mentees were invited to participate in the research surveys; 477 mentees completed the baseline survey (76% response rate). Forty-six percent of the mentor participants were paired with one mentee, 33% of the mentor participants were paired with two mentees, and 21% of the mentor participants were paired with three or more mentees.

For the current analysis, we assigned participating mentees to 1 of 2 categories: those whose mentors attended the workshop (W +) and those whose mentors did not attend the workshop (W−). Of the 477 mentees who participated in the study, 412 were included in the W + group and 65 in the W− group.

Among the 477 mentees, 14% were Hispanic (*n* = 67). Regarding race, 56% were White (*n* = 266), 25% Asian (*n* = 120), 8% African American (37), 6% others (*n* = 28), including American Indian, Hawaiian or unknown and an additional 5% identified with more than one race. About 32% (*n* = 154) identified as being of the first generation to attend college. Approximately 60% were female; 75% were doctoral mentees (versus postdoctoral fellows); and 71% were native English speakers (versus nonnative speakers). The majority of mentees selected basic science as their discipline in the doctoral program (64%), followed by clinical science (25%), population science (8%), and other fields (3%).

Mentees did not attend the workshop. Mentors were not informed of whether their nominated mentees joined the study, and mentees were not required to disclose to their mentor whether they participated in the study. Informed consent was administered; all study procedures were approved by The University of Texas MD Anderson Cancer Center Institutional Review Board, Protocol 2018–0206. Mentees were compensated with a $20 Amazon e-gift code at two time points: upon completion of the pre-survey before their mentor’s scheduled workshop and upon completion of the post-survey 6 months after the workshop.

### Measures

The measures used for the current study were adapted from previous studies (Anderson et al., 2015; Cameron et al., 2020, 2023) to assess the indirect effects of the SCOARE workshop on mentees. These measures were assessed using online surveys administered through REDCap to the mentees at two time points: baseline (i.e., before their mentors were scheduled to attend the workshop) and 6 months after the workshop.

#### Demographic information

Mentees were asked to provide information in a registration questionnaire about their gender (female, male, other, prefer not to answer), race/ethnicity (Hispanic, non-Hispanic, don’t know, and prefer not to answer for ethnicity; American Indian or Alaska Native, Asian or Asian-American, Black or African American, White or European American, other, more than one race, and prefer not to answer for race), whether they were of the first generation to attend college, and academic rank (doctoral or postdoctoral).

In the study questionnaire, to explore potential impacts of home language variety (standardized English [STE] or non-standardized English [NSTE], or English as an additional language [L2]) on the theoretical constructs studied, we first asked if the respondent considered themselves a native speaker of English, and then asked those who indicated English as a native language whether the variety of English they were raised speaking differed from what is typically spoken in the research environment (i.e., standardized English), following a procedure established in a prior study (Cameron et al., 2020). “Standardized English” is the term used by sociolinguists to denote the prestige variety of English and is usually defined as the type of English that is spoken on national news broadcasts. A separate 2023 pre-/post study of SCOARE W + mentees focused explicitly on language variety found that while all mentees whose mentors attended the workshop had significant gains in productivity in speaking tasks, those mentees reporting high language discomfort rated the quality of communication with their mentor and their enthusiasm about communicating significantly higher (*p* < 0.05 for both measures) compared to mentees with low language discomfort. In addition, mentees raised speaking non-standardized varieties of English reported significant reductions in discomfort related to language use (*p* = 0.003), compared to mentees raised speaking standardized English (Cameron et al., 2023).

#### Frequency of communicating

Mentees were asked to report how often they had spoken up, e.g., made comments in discussions or asked questions (“frequency of speaking”) and how often they had been working on scholarly writing projects (“frequency of writing”) during the past 6 months, in both the baseline survey and 6-month post-workshop survey. The response option for these two questions used a 7-point scale, ranging from 1 (never) to 7 (every day).

#### Scientific writing and speaking self-efficacy

A 22-item scale was used to assess mentees’ confidence in their ability to complete specific SC tasks, including scientific writing (10 items) and presentation and informal speaking (12 items; Anderson et al., 2015). Response options were based on a 5-point Likert-type scale, ranging from 1 (not at all confident) to 5 (very confident). Examples of writing and speaking items are “Excel in scientific writing tasks, e.g., abstracts, manuscripts”, and “Effectively answer questions from the audience at a scientific meeting.” A scale score for writing was calculated by averaging 10 items and a scale score for speaking was calculated by averaging 8 items. These scale scores were used for analysis. The Cronbach’s alpha coefficient was 0.86 for writing self-efficacy for both the baseline and post-workshop surveys. For speaking self-efficacy, the coefficient was 0.89 for the baseline and 0.90 for the post-workshop surveys.

#### Science identity

Chemers’ 6-item science identity scale was used to assess the extent to which mentees identified as a scientist on a 5-point scale, ranging from 1 (strongly disagree) to 5 (strongly agree) (Chemers et al., 2011). The Cronbach’s alpha coefficients for baseline and post-workshop surveys were 0.86 and 0.89, respectively. An example item is “I have a strong sense of belonging to the community of scientists.”

#### SC outcome expectations

A 6-item scale was used to assess consequences the mentees expected to result from scientific writing and speaking, on a 5-point scale ranging from 1 (strongly disagree) to 5 (strongly agree) (Anderson et al., 2015). The Cronbach’s alpha coefficients were 0.81 and 0.84 for the baseline and post-workshop surveys, respectively.

#### Reconsideration of a research career due to SC

Mentees were asked to rate their agreement with the statement, “Thinking about the writing and speaking necessary to be successful makes me reconsider my goal of pursuing a research career”, using a 5-point scale ranging from 1 (strongly disagree) to 5 (strongly agree), at baseline and post-workshop. The purpose of this item, used in previous studies, was to determine whether the need to acquire SC skills was perceived as a contextual barrier to career progression by mentees. Reconsideration of career due to the SC required has been found to be associated with speaking both non-standardized native and L2 varieties of English (Trachtenberg et al., 2018).

#### Research career intention

Based on prior research (Cameron et al., 2020), two questions were used to assess mentees’ intention to pursue a career in research using a 5-point Likert scale, ranging from 1 (strongly disagree) to 5 (strongly agree), at baseline and post-workshop. The first question was designed to assess mentees’ intention to lead research, and the second question assessed their intention to pursue a science career without engaging in research activities. For the 2018 cohort, the research career intention questions were not collected at both time points, and intention responses from that group were excluded from analysis. Thus, responses from 2019 to 2021 were included for the analysis (*n* = 374).

### Data analyses

Before conducting the main analyses, Little’s MCAR tests (Little, 1988) were conducted to evaluate patterns of missingness. Additionally, differences in baseline scores were evaluated based on the mentor’s attendance status (W + vs. W−) and across demographic groups. For the main analyses, multilevel modeling (MLM) was performed using a 2-level model: mentees at level 1 and mentors at level 2, with baseline scores included as a covariate. MLM was performed using PROC MIXED in SAS (version 8.2) to estimate the impact of the mentor SCOARE workshop on SCCT and related outcomes among mentees, including SC self-efficacy, science identity, frequency of engaging scientific speaking and writing, SC outcome expectations, and research career intentions. To examine workshop effects, we estimated 3 nested models to identify the best-fitting model, starting with an unconditional model with only an intercept, followed by a model that included a baseline score as a fixed effect, and then a model with the status of mentors’ workshop attendance. We used several fit indices, such as the Akaike information criterion (Bozdogan, 1987), Akaike information criterion for small samples (Bozdogan, 1987), and Bayesian information criterion (Schwarz, 1978), to determine the best-fitting model. We used maximum likelihood estimation to estimate the model parameters and grand mean centering for the predictor variables. Outcomes were transformed to z-scores, following the methodology recommended by Enders and Tofighi (Enders & Tofighi, 2007).

To explore whether the effects of the SCOARE workshop on mentees’ outcomes differed by demographic characteristics, we conducted additional sets of multilevel models only for mentees of mentors who attended the workshop (W+ condition). Each demographic characteristic was examined by adding it as a predictor along with baseline scores as a covariate in the model. In all statistical analyses, *p* < 0.05 was considered statistically significant.

## Results

### Preliminary analyses

The results of the MCAR tests showed no significant association between mentees’ demographic characteristics and missing data with regard to gender (*χ*^2^ [12] = 3.91, *p* = 0.99), rank (*χ*^2^ [12] = 3.88, *p* = 0.99), under- or well-represented status (*χ*^2^ [12] = 3.85, *p* = 0.99), first-generation status (*χ*^2^ [12] = 3.81, *p* = 0.99), home language variety (χ^2^ [12] = 3.90, *p* = 0.99), and status of mentor’s workshop attendance (χ^2^ [12] = 3.84, *p* = 0.99). The Intra-class Correlation Coefficient (ICC) for each outcome ranged around 0.1, indicating that around 10% of the total variance in the outcome variable could be attributed to differences between mentors, thus justifying the use of multilevel modeling to adequately account for the hierarchical structure of the data (Peugh, 2010).

### Workshop effects (W + vs W−)

To answer our first research question (“Is the SCOARE workshop effective in improving mentee outcomes?”), we compared scores of participants in the 2 conditions, W + and W−. Baseline scores showed no significant differences between the W + and W− conditions.

**Fig. 2:**
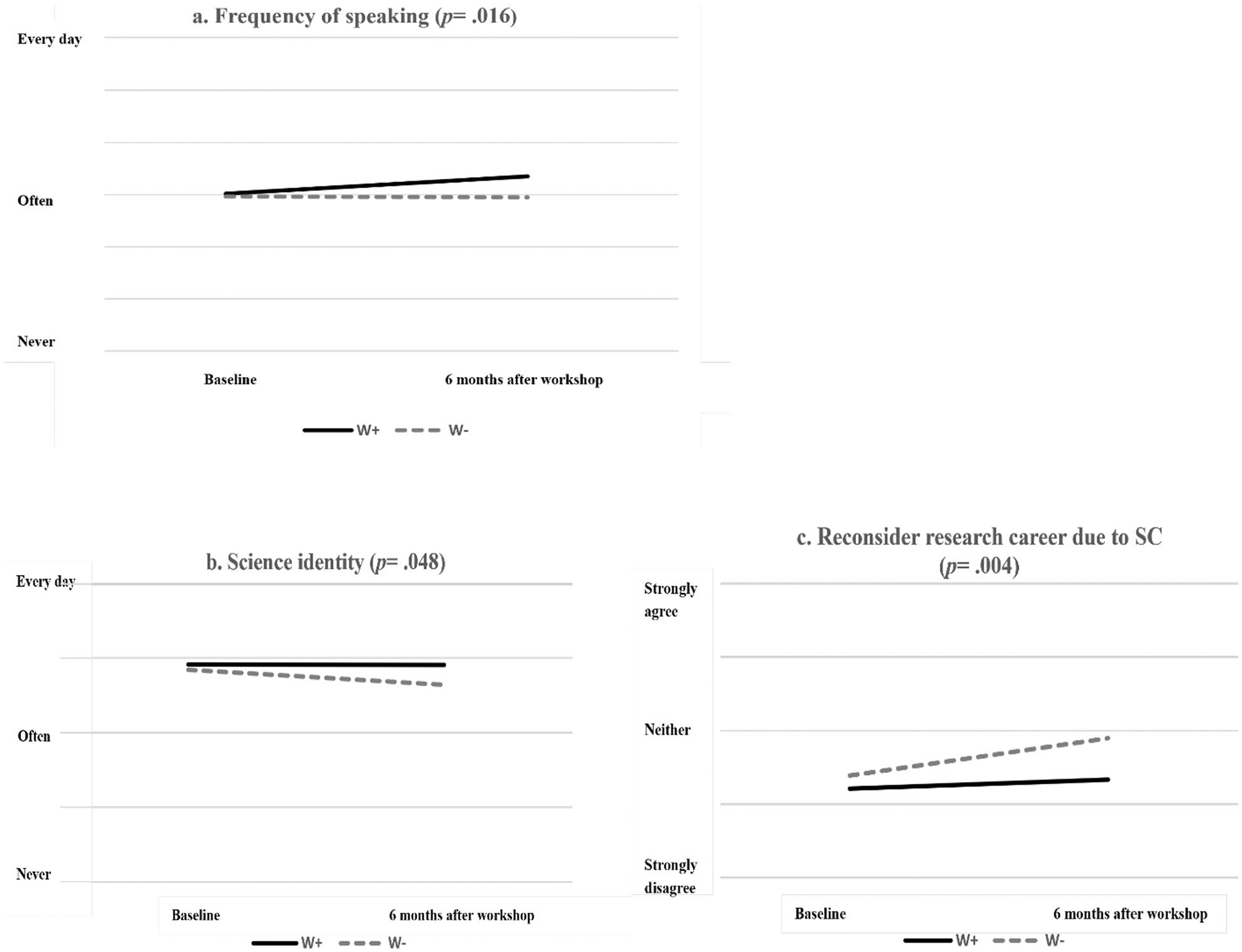
**a–c** Comparisons are made between mentees whose mentors attended the SCOARE workshop (W +) and those whose mentors did not attend (W−), in terms of **a** frequency of speaking, **b** science identity, and **c** reconsideration of a research career due to concerns about scientific communication

Table 1 provides a summary of the MLM analyses used to examine whether mentees’ outcomes for the model’s constructs differed by the status of mentors’ workshop attendance. As shown, the full models, which included baseline scores and the mentors’ attendance status of the SCOARE workshop as predictors, were the best-fitting models for frequency of speaking tasks, science identity, and reconsideration of a research career due to SC skills, based on changes in χ^2^ statistics and fit indices. For frequency of speaking, adding mentor attendance status of the SCOARE workshop improved model fit, with an associated standardized parameter estimate of 0.30 standard deviations more than those in the W− condition, after adjusting for baseline scores (*β* = 0.30, *p* = 0.016; see Fig. 2a). For science identity, adding workshop attendance also improved model fit, with an associated standardized parameter estimate of 0.20 standard deviations (*β* = 0.20, *p* = 0.048; see Fig. 2b). This indicated that science identity of mentees in the W + condition remained stable compared to mentees in the W− condition, whose scores declined. Mentees in the W + condition, after adjusting for baseline, were significantly less likely to report reconsideration of a research career due to SC (*β* = − 0.39, *p* = 0.004; see Fig. 2c) compared to those in the W− condition, whose scores for career reconsideration increased over the 6 months.

Contrary to expectations, after adjusting baseline scores, frequency of writing, writing self-efficacy, speaking self-efficacy, SC outcome expectations, and research career intentions did not differ significantly by mentor workshop attendance.

### Differences in SCCT variables by demographics

To answer our second research question (“Do some sub-groups in the W + condition experience SCOARE effects differently than others?”), we compared scores of participants in four categories: gender, parental education, racial/ethnic representation, and language variety. There were no significant differences in mentees’ outcomes associated with gender, parental education, or well/underrepresented status, indicating similar patterns within each of these paired group comparisons.

### Differences associated with stage of training

After adjusting for baseline scores, postdoctoral mentees, compared with doctoral mentees, scored higher for science identity (*β* = 0.22, *p* = 0.02; Table 2) and lower for intention to pursue a non-research-intensive career (*β* = −0 .33, *p* = 0.004; Table 2). These findings are consistent with the greater length of time in training and the commitment postdoctoral mentees have already made to remaining in research.

### Differences associated with language variety

Language variety was associated with race and ethnicity, as expected, due to its role as a marker of social identity. Among language-variety groups, 76% of the White mentees indicated that they were raised with STE, whereas 12% indicated NSTE. NSTE was indicated by 30% of the Hispanic/Latino/a mentees, 33% of the Asian-American mentees, and 58% of the African American mentees. L2 was indicated by 57% of the Asian-American mentees, 41% of the Hispanic/Latino/a mentees, 13% of the African American mentees, and 12% of the White non-Hispanic mentees.

After adjusting for baseline scores, career reconsideration due to SC concerns was comparatively higher in both the NSTE and L2 groups (*β* = 0.40, *p* = 0.001; *β* = 0.41, *p* < 0.001; Table 3), indicating that scores for both time points were higher in the NSTE and L2 groups than in the STE group.

Regarding intention to pursue a non-research-intensive career, the NSTE group scored higher than the STE group (*β* = 0.35, *p* = 0.003; Table 3), while the L2 group no significant difference to the STE group.

## Discussion

The present study extends previous research on mentor training in scientific communication skills by examining the effects of mentor training on mentees. Participation of mentors in a 6-h evidence-based workshop intervention for mentoring SC skill development results in meaningful benefits for mentees 6 months after the intervention. Mentees with SCOARE-trained mentors reported increased frequency of speaking and the preservation (as opposed to decline) of their science identity, both found in previous studies to be predictive of intent to pursue a research career, as well as lower likelihood of reconsidering a research career due to SC concerns than the W− group.

These results are consistent with our prior findings regarding the role of SC skills and mentoring behaviors in fostering science identity. Frequency of speaking is largely overlooked in studies of science identity, although speaking up has been proposed as an important tool for developing both self-recognition and recognition by others, foundational building blocks of science identity (Carlone & Johnson, 2007; Hudson et al., 2018; Malone & Barabino, 2009). The SCOARE study findings provide empirical evidence for this view. Science identity has received substantial attention in studies of undergraduates or graduate students and is a frequently-cited precursor of research career intention (Chemers et al., 2011; Estrada et al., 2011; Hernandez et al., 2013; Stets et al., 2017; Byars-Winston & Rogers, 2019). Recent studies have found science identity to be relevant at the postdoctoral level, and related to career persistence (Hudson et al., 2018; Thakar et al., 2023). Results of the current study support the relevance of science identity at the doctoral and postdoctoral stages of training and its ability to be modified through a relatively simple intervention even at the later stages of training.

Mentee thoughts of reconsidering a research career due to concerns about the need to engage in scientific communication held steady after mentors attended the SCOARE workshop, while they increased for those whose mentors did not attend. This outcome provides additional insight into the degree to which SC skills affect the dynamic process of mentee career considerations over time and that perceptions about SC skills may be a potential point of vulnerability in persistence in research-intensive career paths. Although many factors influence career choice and persistence among STEM research trainees, the SCOARE intervention affords a concrete, actionable way to potentially mitigate mentee stress in one aspect of their training experience.

The positive effects for mentees resulted without their active participation in the intervention, highlighting the downstream impact that an intervention for mentors can have on mentees. SCOARE evaluation outcomes (Dahlstrom et al., 2022) and personal mentor communications suggest that even modest application of the techniques for demandingness and responsiveness in SC may be the mechanism driving results. Recent studies of mentor-training effects have shown that such interventions can result in increased mentor self-reported use of mentoring skills (Antonio et al., 2004; Johnson, 2015; Pfund et al., 2006). With respect to mentee outcomes, Pfund et al. (2014) found that mentees reported receiving mentoring behaviors more often after their mentors participated in a mentoring workshop. Measuring impact of educational interventions on mentees or students involves many complexities, but studies such as those of Hecht et al. (Hecht et al., 2023) and Asher et al. (Asher et al., 2023) provide evidence that even brief interventions can produce meaningful results for students. While the effect of the SCOARE intervention, with betas ranging from 0.20 to 0.39, may seem modest compared to results of controlled studies, such effects are considered meaningful for uncontrolled field studies and for studies collecting data several months post-intervention. Reviews of field intervention effect sizes note that effect sizes of 0.10–0.20 *SD* represent substantial impact (Chetty et al., 2014; Hill et al., 2008; Kraft, 2020; Yeager & Walton, 2011). The SCOARE study is significant because of its finding that a workshop intervention can produce lasting social-cognitive and behavioral effects for mentees, including higher frequency of speaking, science identity, and lower career reconsideration due to SC at posttest. These outcomes are interesting and important, as they underscore the power of linguistically informed, trained mentoring.

Contrary to expectation, significant differences between the W + and W− groups were not found across all of the SciComm SCCT components, including frequency of writing, writing self-efficacy, speaking self-efficacy, SC outcome expectations, and career intention. The absence of change in these constructs could be due to a need to extend workshop content and/or contact hours. It is also possible that six months is not a sufficient time span to observe or effect changes. Writing opportunities, in particular, are often determined by external circumstances such as availability of experimental results, which limits the possibility of increasing writing frequency. Frequency of informal speaking (in meetings or when networking, etc.), on the other hand, can occur freely and even daily and is easier for mentors to encourage and stimulate; this activity did increase significantly. Our prior empirical model indicated that certain SC-related social-cognitive career-theoretical variables predict career intentions both directly and indirectly. Although the current study found no significant difference in SC self-efficacy and research career intentions among trainees between W + and W− groups, it does indicate that SCOARE helps in enhancing the upstream factors that influence career intentions (frequency of speaking, SC mentoring received, and science identity). In addition, the finding that career reconsideration due to SC concerns did not increase for the W + group, while it did increase for the W− group, suggests that SCOARE may help ameliorate that particular barrier to career intention. In sum, our findings emphasize malleable factors among mentees that can be addressed in brief, targeted, mentor training, serving as a practical and positive investment for mentors, mentees, and educators.

Regarding the exploratory findings for outcomes among different subgroups including gender, parental education, racial/ethnic representation, and language variety, only the language variety subgroups showed differences in career reconsideration due to SC concerns, with the NSTE and L2 groups reporting greater reconsideration than the STE group both at baseline and after the workshop intervention. The results suggest that home language variety may influence mentee perceptions of their own SC skills and what those skills mean for their career progression; this is consistent with the earlier finding that NSTE and L2 mentees experienced reduced discomfort due to language background after their mentors participated in the workshop, while no pre-/post-effect was found for STE mentees, whose comfort level is high to begin with. In other words, speakers of standardized English may not perceive linguistic background as a barrier in the way that non-standardized dialect speakers and L2 speakers do, adding to the evidence that linguistic background may be an issue for equity and inclusivity in the STEM research workforce and deserving of greater attention. Further research is needed to determine whether a more tailored intervention might be helpful to address language variety perceptions. These novel findings highlight the potential role of linguistic background as a factor in research career outcomes and demographic representation, a topic which could inform development of interventions for diversity, equity, and inclusion.

The SCOARE workshop does not focus on teaching mentors to improve the “quality” of writing and speaking directly; it is designed instead to provide mentors with productive, encouraging approaches and behaviors that foster mentee behavioral engagement with SC tasks, which in turn helps strengthen mentees’ identity as scientists. Hecht et al. (2023) reported that high school students maximized their achievement gains when their teachers reported using a growth mindset approach characterized by providing high support, establishing high standards, and using strategies such as global encouragement, substantive feedback, and wise feedback that conveys high expectations and confidence in the learner’s ability to progress. The SCOARE approach is closely aligned with these principles and practices. Although the Hecht study occurred in a different educational setting, we speculate that the positive effects of the SCOARE workshop may be a result of actively promoting attitudes reflecting inclusivity and a growth mind set among mentors.

### Limitations

We note some limitations of the current study. The size of the W− group is substantially smaller than that of the W + group, as the W− group is not a true control condition. Although mixed-effects models such as used for this study are well-suited to handle data with uneven group sizes, the possibility remains that significant results, which might have emerged with a larger W− group, could be overlooked. In addition, some bias may have been introduced by the fact that for both W + and W− groups, mentors had expressed interest in the study and had already invited mentees to join the study. It is possible that the initial willingness of both groups of mentors to participate reflects a group of mentors who are already interested in working on SC skills, such that the mentoring experience of mentees in both groups in the study may differ from that of the general mentee population. However, the fact that the mentees of the non-participating but interested mentors exhibited significantly different outcomes from those whose mentors did participate supports the finding that the techniques and insights learned in the workshop lead to new, beneficial behavioral and attitudinal changes in mentees. Finally, although findings suggested that mentees who grew up with NSTE and L2 backgrounds had higher career reconsideration due to SC concerns than those with STE backgrounds as well as greater intention to pursue non-research-intensive careers, a larger comparison group could help confirm these results as well as reveal additional subgroup differences, whether of language variety groups or others, such as stage of training.

### Implications for mentoring practice

The SCOARE workshop’s underlying strategy is to shift the mentoring mindset from thinking of communication as a procedural, cognitive skill that must be performed “correctly” into thinking of its broader, more consequential possibilities as a driver of identity and belonging that mentors can *develop and foster*—process over product. Acquiring scientific language does not require the mentee to relinquish their home variety; it is an added skill that the speaker can choose to use when desired. Many mentors view this approach favorably; beneficial effects can be brought about through accessible and immediately applicable techniques that do not require intensive training in writing and speaking pedagogy. Many attendees have described using the SCOARE approach as more rewarding and less time-consuming than their traditional approaches and have commented that they learned new strategies for building their own communication skills as well, an additional benefit.

Because the SCOARE workshop trains mentors rather than mentees, the effects may be generative, because the mentor will presumably work with multiple mentees over the course of their career. Many of the mentees whose mentors received SCOARE training are themselves future mentors and training program directors and may model their own mentoring approach in a similar way. To this end, a version of SCOARE for postdoctoral trainees has recently been introduced.

### Future directions

The next steps for this program of research include analysis of mentor behaviors and perceptions following participation in the workshop and exploration of SCOARE strategies. This will provide insights into ways to tailor the workshop curriculum for greater efficiency and effectiveness and to help participants focus their energies. Longitudinal follow-up with mentees may shed light on SC behavioral, social-psychological, and attitudinal impacts over the long term, and further exploration of the role of language variety as a dimension of identity may help illuminate its potential for promoting inclusivity not only in the STEM research environment but also more generally.

## Conclusion

Feasible, focused, and effective approaches for faculty mentors to improve the professional skills of their mentees will help build the STEM research workforce. Mentoring SC skills has a variety of positive social-psychological, behavioral, and practical impacts on STEM research mentees and is teachable. Application of SC mentoring skills as an important component of a general mentoring plan can help provide mentees with a more satisfying research training experience as well as equip them for a wide variety of careers.

## Figures and Tables

**Table 1:** Values show the impact of mentor attendance (W + or W−) at the SCOARE workshop on their mentees’ outcomes

	Frequency of speaking		Science identity		Reconsideration of research career due to SC	
	β	SE	β	SE	β	SE
Intercept	− 0.26	0.12	− 0.17	0.09	0.35	0.12
Baseline	0.36	0.03	1.00	0.05	0.321	0.03
W + vs W−	**0.30** *	0.12	**0.20** *	0.10	− 0.39**	0.13
Variance components						
Intercept	0.00	0.05	0.02	0.03	0.02	0.05
Residual	0.74***	0.07	0.45***	0.04	0.79***	0.07

Note:

* *p* < .05.

** *p* < .01.

*** *p* < .001

Bolded values indicate statistically significant outcomes values

**Table 2:** Differences in mentee outcomes by stage of training within W +

	Science identity		Intention to pursue a non-research-intensive career	
	*β*	SE	*β*	SE
Intercept	− 0.04	0.04	0.09	0.05
Baseline	0.97***	0.05	0.60***	0.04
Postdoctoral vs doctoral (ref)	**0.22** *	0.09	− 0.33**	0.11
Variance components				
Intercept	0.00	0.03	0.02	0.05
Residual	0.49***	0.05	0.54***	0.06

Note: W + indicates mentees whose mentors attended the SCOARE workshop.

* *p* < .05.

** *p* < .01.

*** *p* < .001

Bolded values indicate statistically significant outcomes values

**Table 3:** Differences in mentee outcomes by language variety within W +

	Reconsider research career due to scientific communication		Intention to pursue non-research intensive career	
	*β*	SE	*β*	SE
Intercept	− 0.19***	0.07	− 0.07	0.06
Baseline	0.27***	0.04	0.59***	0.04
NSTE vs STE (ref)	**0.40** **	0.12	**0.35** **	0.12
L2 vs STE (ref)	**0.41** ***	0.12	0.04	0.11
Variance components				
Intercept	0.00	0.00	0.00	0.05
Residual	0.79***	0.06	0.56***	0.07

W + indicates mentees whose mentors attended the SCOARE workshop

*NSTE* non-standardized English, *STE* standardized English, *L2* non-native English

* *p* < .05.

** *p* < .01.

*** *p* < .001

Bolded values indicate statistically significant outcomes values

## Data Availability

The datasets used and/or analyzed during the current study are available on Open Source Framework at SCOARE Project OSF.
